# The Complexity of Immunoglobulin A Immune Responses in Respiratory Syncytial Virus Infection

**DOI:** 10.3390/v18020150

**Published:** 2026-01-23

**Authors:** Ashley Ferrier Esposito, Diego R. Hijano, Stephania A. Cormier

**Affiliations:** 1 Department of Biological Sciences, Louisiana State University, Baton Rouge, LA 70802, USA; aesposito1@lsu.edu (A.F.E.); stephaniacormier@lsu.edu (S.A.C.); 2 Pennington Biomedical Research Center, Baton Rouge, LA 70808, USA; 3 Department of Infectious Diseases, St. Jude Children’s Research Hospital, Memphis, TN 38105, USA; 4 Department of Pediatrics, University of Tennessee Health Science Center, Memphis, TN 38103, USA

**Keywords:** mucosal immunity, respiratory syncytial virus, immunoglobulin A, B cells, regulatory B cells

## Abstract

Respiratory syncytial virus (RSV) remains a leading cause of severe lower respiratory tract disease in infants worldwide. Despite extensive study in animal models and humans, fundamental age-dependent differences in mucosal immunity continue to limit the development of durable protective strategies in early life. Compared to adults, infants mount weaker humoral responses to RSV, underscoring the urgent need for effective vaccines in this age group. Immunoglobulin A (IgA), the dominant antibody isotype at respiratory mucosal surfaces, plays a central role in limiting viral replication and disease severity during RSV infection. While IgA limits RSV severity in adults, infants fail to generate robust IgA responses. Impaired IgA responses in infancy reflect unique immune regulatory pathways that shape early-life antiviral immunity. Emerging evidence highlights a critical role for regulatory B cells (Bregs), particularly neonatal Bregs (nBregs), in suppressing antiviral responses, limiting class switch recombination, and contributing to severe RSV disease. This review summarizes current evidence on IgA regulation during RSV infection, with particular emphasis on age-specific B-cell responses and the emerging role of Bregs. Improved understanding of these mechanisms has direct implications for the rational design of vaccines and immunomodulatory strategies tailored to infants.

## 1. Introduction

Respiratory syncytial virus (RSV) is a highly contagious respiratory pathogen that poses significant health risks for infants and the elderly [1,2,3]. A member of the *Pneumoviridae* family, RSV is an enveloped, negative-sense, single-stranded RNA virus of approximately 15,000 base pairs that encodes 11 proteins essential for viral fusion, entry, replication, and assembly. Key proteins include the attachment glycoprotein (G) and fusion glycoprotein (F), which mediate viral attachment and entry, as well as the nonstructural proteins NS1 and NS2, which antagonize host antiviral immune responses [4,5]. Other proteins include the nucleocapsid (N), phosphoprotein (P), matrix protein (M), two transcription regulatory proteins (M2-1 for transcription processivity and M2-2 for viral RNA replication), and a large polymerase (L), which aids in viral assembly. Additionally, RSV has small hydrophobic (SH) proteins expressed on its surface. The role of this accessory protein is poorly understood but appears to be crucial for RSV’s virulence by inhibiting TNF-α-mediated apoptosis [6,7,8].

RSV is among the leading causes of early childhood viral infection worldwide. In the United States, RSV leads to 58,000–80,000 hospitalizations, with 500,000 emergency department visits and 1.5 million outpatient clinic visits, leading to 100–300 deaths per year in children younger than 5 years of age [9,10,11,12,13,14,15,16,17]. The burden is greatest among infants under six months of age, particularly those with prematurity, congenital heart or lung disease, or immunocompromise [18].

Clinically, RSV infection ranges from mild upper respiratory tract illness to severe bronchiolitis and pneumonia, with complications including hypoxemia, apnea, and respiratory failure. Severe infant RSV is also linked to long-term consequences such as recurrent wheezing and asthma that persist in adulthood [19,20,21,22,23,24].

Despite decades of research, the development of a safe and effective infant RSV vaccine remains a major challenge. The use of formalin-inactivated RSV in the 1960s caused enhanced disease in vaccinated children, highlighting the complexity of RSV immunity [25]. Recently, three vaccines (Arexvy^®^, Abrysvo^®^, and mRESVIA^®^) have been approved for older adults, with Abrysvo^®^ also administered during pregnancy to protect newborns during their first months of life. In addition, the long-acting monoclonal antibodies Nirsevimab and Clesrovimab are now recommended for infants to prevent severe RSV during their first season. However, these strategies do not provide long-term immunity, and the immune response in infants remains poorly understood

This review focuses on mucosal IgA responses to RSV, emphasizing their unique features in infancy. We discuss current knowledge of B-cell development, class switching, and the role of IgA, highlight the emerging role of regulatory B cells (Bregs) in RSV pathogenesis, and outline gaps that must be addressed to inform vaccine and therapeutic development for infants.

## 2. Pathogenesis of RSV

RSV pathogenesis reflects complex virus–host interactions, with disease severity influenced by age and comorbidities. The virus spreads primarily through droplets from coughing or sneezing, direct contact with secretions, or contaminated surfaces, where it can remain infectious for hours [26,27]. The incubation period ranges from 2 to 8 days, after which infection typically begins in the upper respiratory tract. Within a few days, RSV can extend to the lower respiratory tract via aspiration or by fusion of epithelial cells, forming syncytia, a phenomenon primarily observed in cell culture systems [28].

RSV infection is most severe in infants, particularly those younger than six months. Up to 18% of infants under one year of age experience apnea early during RSV infection [29,30]. Both human and animal studies demonstrate that RSV infection early in life is associated with long-term pulmonary consequences, including persistent airway dysfunction [23,24,31,32,33,34,35,36].

## 3. RSV Immune Response

The host immune system plays a pivotal role in protecting against and recovering from RSV infection. Various components contribute to this defense, including maternally derived antibodies in infants, host-derived antibodies, and T-cell responses [28,37,38,39,40,41]. Both CD4^+^ and CD8^+^ T cells play crucial roles in the immune response to RSV infection. CD4^+^ T helper (Th) cells release cytokines important in facilitating the humoral immune response, including B-cell activation, antibody production, and antibody class switching [42] in addition to activating CD8^+^ T cytotoxic (Tc) cells [43,44]. Th cells can be subdivided into several subtypes. The major subtypes studied in RSV include Th1, which releases interferon-γ (IFN-γ), and Th2, which releases interleukins (IL) 4, 5, and 13 [45,46,47,48,49,50,51,52]. CD8^+^ Tc cells expressing IFN-γ play a crucial role in controlling and clearing RSV-infected cells and providing long-term immunity [53,54], while Th2 cells have been shown to promote pathogenesis, leading to increased mucus production and airway hyperreactivity [55,56,57].

Infants receive passive immunity against RSV from maternal antibodies transported through the placenta before birth and through breastmilk for the first few months of life, when the infant immune system is still immature [58,59,60,61,62,63]. These antibodies, which include IgG and IgA, play a critical role in protecting infants against RSV infection. Interestingly, RSV elicits weak secretory antibody responses in infants [28,64,65], and it is believed that this is due to the immaturity of the infant immune system and/or a suppressive effect of maternally transmitted antibodies [66,67].

## 4. B Cells in RSV

B-cell development is critical during RSV infection. Depletion of B cells impairs CD4^+^ T-cell priming, which affects the activation and clonal expansion of T cells, leading to a higher viral load and delayed clearance [68]. Human B-cell development involves a cascade of cellular events that begins in the fetal liver and bone marrow. The purpose of B-cell development is the generation of a plethora of immunoglobulin molecules via extensive selection processes, which, by virtue of mutation and gene recombination mechanisms, confer diverse immunity against a variety of pathogens [69].

Class switch recombination (CSR) in B cells is a vital process in the adaptive immune system. It involves the rearrangement of the constant region of the antibody gene, resulting in the production of different classes of antibodies [70]. Antibody classes tailor the immune response to combat different types of pathogens, and each has distinct roles in immune defense and inflammation.

In mucosal immunity, IgA plays a critical protective role. In the respiratory system, IgA-secreting plasmablasts are recruited to the mucosal epithelium via CCR10 interaction with the CCL28 chemokine produced by respiratory epithelial cells [71]. IgA on mucosal surfaces appears to be important against RSV, although its full role in RSV pathogenesis remains unclear. Studies have shown a positive correlation between RSV-specific IgA and protection against disease [72]. However, unlike adults, human infants (hereafter referred to as ‘infants’) and neonatal mice (hereafter referred to as ‘neonates’), both of which develop more severe disease, fail to mount strong IgA responses [73,74,75]. Consistent with human data, we previously observed a strong correlation between the production of IgA and type-I interferons (IFN-I) [73,76,77,78]. IFN-I mediates antiviral immunity by promoting inflammation and recruiting immune cells essential for viral clearance [79]. RSV induces robust IFN-I responses in adults but not in infants, underscoring the age-dependent nature of immune responses to RSV [80,81,82,83]. This phenomenon can be partially explained by the underdeveloped immune response, with a dominant anti-inflammatory response in infants, and the role of the viral proteins NS1 and NS2, which can block IFN-I responses [5,81]. Adults can counteract and overcome the antagonism of the NS proteins and mount a strong antiviral response.

Among IFN-I, IFN-α is particularly important for shaping the immune response to RSV [84,85]. We have shown that nasal-associated lymphoid tissue (NALT) from RSV-infected adult mice contains significantly more IgA^+^ B cells than NALT from neonates [73]. Strikingly, this difference disappears when neonates receive IFN-α prior to RSV infection, highlighting IFN-α’s role in B-cell activation and IgA production [73]. In addition, neonates infected with RSV and receiving IFN-α produced significantly higher levels of B-cell activating factor (BAFF), a TNF family member that promotes B-cell development, compared to untreated littermates [73,86]. Collectively, these findings indicate that age and IFN-α critically influence B-cell activation, differentiation, and IgA-mediated immunity against RSV infection and re-infection.

**Figure 1:**
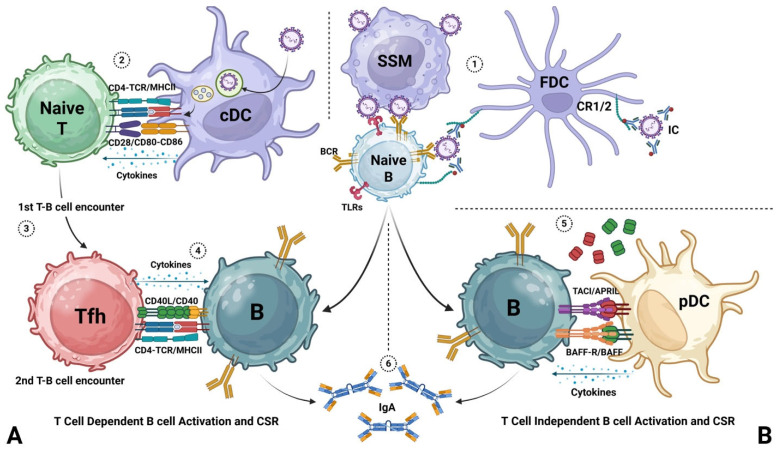
**T-cell-dependent and T-cell-independent B-cell activation and class switch recombination.** (**A**,**B**) T-cell-dependent activation. (1) In secondary lymphoid organs, subcapsular sinus macrophages (SSM) capture antigen and deliver it to follicles. Antigen immune complexes bind complement receptors on follicular dendritic cells (FDCs) and are presented to antigen-specific B cells in a sustained manner. (2) In the T-cell zone, conventional dendritic cells (cDCs) present antigen to naïve CD4^+^ T cells in an MHC II-dependent context. (3) Primed T cells migrate toward the B-cell follicle and engage primed B cells presenting the same epitope via MHC II. ICOS-ICOSL interactions support differentiation into follicular helper T cells (Tfh) and initiation of the germinal center reaction. (4) Within germinal centers, B cells undergo clonal expansion, somatic hypermutation, affinity maturation, and class switch recombination (CSR). CD40-CD40L interactions and Tfh-derived cytokines promote CSR and the generation of plasmablasts, plasma cells, and memory B cells. T-cell-independent activation. (1) T-cell-independent priming is driven by nonprotein antigens that cannot be presented via MHC II, including polysaccharides, lipids, and nucleic acids. These antigens can crosslink multiple B-cell receptors (BCRs) and trigger pattern recognition receptor signaling, including Toll-like receptor pathways. (2) CSR can be induced through interactions with plasmacytoid dendritic cells (pDCs) and other antigen-presenting cells that express BAFF and APRIL, engaging BAFF-R and TACI, together with cytokine signals such as type I interferons, IL-10, and TGF-beta. (3) IgA can be generated through both pathways. However, T-cell-independent responses typically produce lower-affinity antibodies and do not generate durable memory because affinity maturation is limited.

B-cell activation and subsequent CSR are categorized based on stimuli and consist of T-cell-dependent (TD) and T-cell-independent (TI) responses [87,88]. In both processes, naïve B cells first require an activation signal, antigen binding to a B-cell receptor (BCR). TD B-cell development [89,90] (Figure 1) typically occurs in secondary lymphoid organs, such as lymph nodes or the spleen. Antigens are captured by dendritic cells, processed, and presented via MHC II to naïve CD4^+^ T cells in the T-cell zone. Upon antigen recognition, primed T cells migrate toward the B-cell follicle for the initial T–B-cell encounter. This crosstalk, mediated by ICOS–ICOSL interactions, induces the differentiation of follicular helper T (Tfh) cells. These cells migrate to the germinal center, where they stimulate B cells through cytokine secretion and engagement of CD40–CD40L interactions. These signals induce class switch recombination and affinity maturation. This process can occur repeatedly, resulting in the progressive selection of antibodies with increased affinity. Class switch recombination (CSR) involves a change in the Ig class produced by B cells, resulting in the production of antibodies with diverse effector functions [70,91]. Tfh-derived cytokines, including IL-21, IFN-γ, TGF-β, IL-2, IL-4, IL-5, and IL-10, help determine B-cell fate [92,93,94,95,96,97,98,99]. Following interaction with Tfh cells, B cells undergo clonal expansion and differentiation. This process leads to the formation of plasmablasts, which are plasma cells capable of producing large quantities of antibody. Some B cells undergo repeated antigen encounters that promote the selection of higher-affinity antibodies. This process, known as affinity maturation, enhances the overall efficiency of the humoral immune response. TD B-cell responses are crucial for generating high-affinity antibodies, the development of immunological memory, and long-lasting protection against pathogens [100].

In TI B-cell development, on the other hand, T-cell help is not required for B-cell activation. TI B-cell development is triggered by antigens that directly bind B-cell receptors and engage pattern recognition receptors, such as toll-like receptors (e.g., TLR4) [101] (Figure 1B). TI B responses typically produce lower-affinity antibodies, as they do not undergo affinity maturation and are less effective at generating long-lasting immunological memory. In TI B-cell development, class switching has been shown to occur through the engagement of BAFF receptors (BAFF-R) and the transmembrane activator and CAML interactor (TACI) with BAFF and a proliferation-inducing ligand (APRIL), which is expressed on the membrane and also secreted by antigen-presenting cells along with IFN-α, IL-10, and TGF-β. Among antigen-presenting cells, plasmacytoid dendritic cells (pDCs) are a major source of IFN-α. Notably, pDC numbers are significantly reduced in the lungs of RSV-infected neonatal mice [102,103,104,105]. Despite growing recent attention, TI B-cell responses to RSV remain poorly characterized.

In vitro studies and studies on post-mortem infant lungs from fatal RSV infections suggest that the B-cell response to RSV infection is primarily driven by the TI pathway [106]. Specifically, infant B cells in culture and infected with RSV express lower levels of co-stimulatory molecules CD40 and CD80/86, decreasing their receptivity to CD40L on T cells [107] and producing low levels of TD cytokines, including IL-2, IL-4, and IL-10 [52,106]. Since antigen presentation due to lack of co-stimulatory molecules is diminished and the number of CD4^+^ T cells is lower in infected infants, the secreted TD cytokines are also reduced, and there is a failure to induce the TD pathway. In post-mortem infant lung tissues, increased levels of BAFF and APRIL were detected in infected epithelial cells, indicative of a TI pathway [106]. In vitro, RSV infection of B cells in culture failed to induce cytokine and antibody production, whereas the addition of BAFF with RSV infection enhanced antibody production by following the TI pathway [106].

## 5. Regulatory B Cells

Antibodies are extremely important to the immune system because of their ability to contribute to an efficient immune response against invading pathogens and neutralize them before they cause severe diseases. In contrast, in cases of immune dysregulation, such as allergic asthma, experimental autoimmune encephalomyelitis, and lupus, production of such antibodies plays a pathogenic role [108,109,110]. Chronic allergic asthma results in airway remodeling due to persistent inflammation. This inflammation can be traced back to pro-inflammatory antibodies from plasma B cells [3]. While they are conventionally believed to contribute to an inflammatory response, a subset of these B cells was recently found to dampen inflammation. These cells are called regulatory B cells or ‘Bregs’. In the context of viral infection, including RSV, Bregs exert immunomodulatory effects that can profoundly shape antiviral immunity, inflammation, and disease severity [111,112]. The regulatory capacity of B cells can be induced by inflammatory signals, including TLR stimulation and proinflammatory cytokines, such as BAFF and APRIL [113,114,115]. Bregs are able to control inflammation by exerting immunomodulatory effects through the secretion of cytokines such as IL-10 and TGF-β, which can dampen the immune response and inhibit the onset of allergic asthma and other inflammatory diseases, thereby reestablishing homeostasis [108,109,110,116,117,118,119,120]. Different subsets of Bregs have been described, and they can be found at different stages of B-cell development. Currently, there is no common marker across the several Bregs described, and the immunosuppressive mechanism exerted by these cells involves different combinations of surface molecules and soluble factors. Table 1 summarizes the most studied Breg subsets. While Bregs contribute to immune homeostasis across multiple inflammatory conditions, their regulatory functions appear particularly consequential during RSV infection in early life, when antiviral immune responses are inherently constrained.

The Breg, TD B-cell, and TI B-cell responses are distinct aspects of the immune system, each defined by unique mechanisms and functions. The initiation of Breg responses is highly context-dependent, influenced by the nature of the immune challenge, the tissue microenvironment, and the specific signals present. Unlike TD responses, Bregs are generally not classified as TD because they can be induced and function without T-cell help, often through signals such as BAFF, APRIL, TGF-β, IL-10, and TLR activation [106,113,151,152,153]. Although Bregs share certain features with conventional B cells involved in TI responses, their roles are fundamentally different. TI responses primarily drive rapid antibody production against specific antigens, whereas Bregs contribute to immune homeostasis and regulation. Nevertheless, Bregs can interact with CD4^+^ T cells and other immune cells through direct contact or soluble mediators. These interactions include inhibiting proliferation and suppressing inflammatory cytokine expression by CD4^+^ and CD8^+^ T cells, which may influence B-cell class switching [154]. Such antigen-dependent crosstalk between Bregs and T cells can affect antibody production and the immune response following RSV infection. A deeper understanding of the diverse mechanisms that initiate Breg responses is essential to elucidate their roles in immune regulation.

## 6. Role of Bregs in RSV

**Figure 2:**
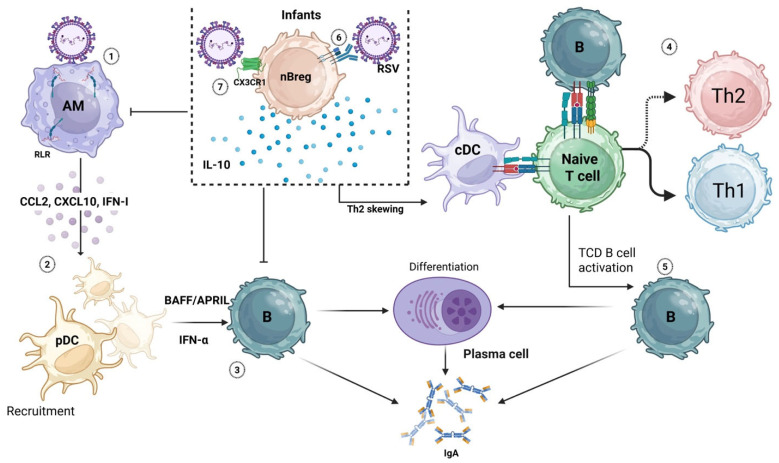
**Potential role of nBregs in immune response against RSV in infants.** (1) In the lung, alveolar macrophages recognize RSV RNA through pattern recognition receptors, including RIG-I-like receptors, and initiate type I interferon responses and chemokine production. (2) These signals promote recruitment of antiviral cells, including plasmacytoid dendritic cells (pDCs), which can express and secrete BAFF and APRIL. (3) These molecules induce TCI B-cell activation and differentiation into plasma cells with production of low-affinity IgA. (4) On the draining lymph nodes, T-cell priming by cDCs together with the crosstalk with B cells results in a Th1 and Tc1 response against the virus, (5) followed by TCD B-cell activation, differentiation, and affinity maturation, which leads to high titers of high-affinity IgA against RSV. (6) In infants, a different scenario emerges; neonatal regulatory B cells (nBregs) can recognize RSV F protein through the B-cell receptor, leading to the upregulation of CX3CR1. (7) CX3CR1 facilitates RSV infection of nBregs through interactions with the RSV G protein, resulting in IL-10 production that is enhanced by the IFN-I produced by AMs. IL-10 can inhibit alveolar macrophage activation and chemokine secretion, reducing the recruitment and activity of pDCs and other antiviral effector cells. IL-10 can also bias T-cell priming away from Th1 and Tc1 responses and toward Th2 and Tc2 responses, which are associated with airway reactivity, mucus production, and structural remodeling. IL-10 itself and these affected mechanisms may limit B-cell activation and class switch recombination, impair viral clearance, and hinder the development of durable mucosal IgA responses and immunological memory.

In the context of RSV, Zhivaki et al. identified a subset of Bregs specific to infants [140]. These cells, called neonatal Bregs (nBregs), are a distinct subset of Bregs that are permissive to RSV infection and share the most common immunosuppressive mechanism, IL-10, but with distinct surface identification markers and with an exclusive developmental window during which they are present in the lungs [139,140]. These nBregs are absent in adults but are present in high frequency in cord blood and early infancy, coinciding with the developmental window of heightened RSV disease severity. Interestingly, they can be infected with RSV, whereby they can produce significant amounts of IL-10. Frequency of nBregs (CD5^+^CD23^hi^) in infants with RSV-induced acute bronchiolitis correlates with higher RSV load and increased disease severity as determined by length of hospital stay, oxygen support, and pediatric intensive care unit admission [140]. The B-cell receptor on these nBregs serves as an attachment point for the RSV F protein, a phenomenon made possible by the intrinsic polyreactivity of the nBreg BCR. More specifically, the IgM component of this receptor exhibits a shorter complementarity-determining region 3 (CDR3) within the immunoglobulin heavy chain variable (IGHV) genes, resulting in a B-cell subset with a distinct repertoire and unique functional characteristics. The BCR recognition leads to upregulation of the receptor CX3CR1, which interacts with the RSV G protein and allows RSV infection, which induces the production and secretion of IL-10 by these cells. Moreover, during RSV infection, first-line immune responders such as alveolar macrophages recognize viral RNA through RIG-I-like receptors and initiate type I interferon responses. In infants, these signals paradoxically amplify IL-10 production by nBregs, creating a regulatory feedback loop that suppresses macrophage activation, dampens the recruitment of immune cells, limits antiviral T-cell responses, and may indirectly impair IgA induction [140,155]. Understanding the functions of Bregs and their roles in diseases such as RSV remains an evolving area of research. RSV infection triggers a complex immune response involving multiple cell types and signaling pathways (Figure 2). One study showed that naïve B cells can differentiate in the presence of APRIL into IL-10-producing IgA^+^ B cells with Breg-like properties, capable of suppressing T-cell and macrophage activity [113]. These Bregs differ from neonatal Bregs (nBregs) in that nBregs are present during the early life stage when IgA is absent, whereas IgA^+^ Bregs are more relevant in adults. However, current evidence is insufficient to determine whether nBregs directly influence IgA production. In neonatal mice, nBregs (CD5^+^CD23^−^) form a short-lived lung-resident population that produces IL-10 following RSV infection, peaking at days 6–8 after birth and declining by day 12 [139]. This timing coincides with the period when RSV infection fails to elicit a type I interferon response, leading to limited immune activation and delayed development of IgA-mediated memory. Whether IL-10-producing nBregs suppress IgA production directly or indirectly remains unclear. Further research is needed to elucidate the mechanisms by which nBregs regulate IgA responses in RSV disease and how these insights could inform therapeutic strategies.

## 7. Concluding Remarks

Despite nearly half a century of research, there are still significant gaps in our understanding of RSV immunopathogenesis, especially regarding age at initial infection, the ensuing immune response, and resulting disease severity in the host. This information is crucial for the development of effective vaccines and treatments for this age group. Ongoing research in our lab is aimed at elucidating the mechanistic relationship between IgA production and protection against RSV infection. Advancements in our knowledge of mucosal B-cell responses may pave the way for the design of safe and effective vaccines that specifically enhance IgA-mediated immunity in infants, offering more effective and targeted approaches to combat RSV infections. Integral to this effort is understanding the role of the much-understudied Breg and/or nBreg cells in the mucosal antibody response to RSV, particularly in infants and young children.

## Figures and Tables

**Table 1:** Phenotype of Breg subsets and main functional molecules in humans and mice.

Species	Subtype	Phenotype	Functional Molecules	Ref.
Human	CD1d^hi^ B10	CD19^+^CD5^+^CD1d^hi^	IL-10	[121]
	Transitional Bregs	CD19^+^CD24^hi^CD38^hi^	IL-10, IL-35 TGF-β	[122,123]
	Memory B10	CD19^+^CD24^hi^CD27^+^	IL-10	[124,125]
	Plasmablast B10	CD19^lo^CD27^hi^CD38^hi^	IL-10	[126,127]
	TIM-1 Bregs	CD19^+^TIM-1^+^	IL-10	[128,129]
	PD-L1 Bregs	CD19^+^PD-L1^+^	IL-10 and PD-L1	[130]
	Fas-L Bregs	CD19^+^CD38^+^IgM^+^FasL^+^	FasL and IL-10	[131,132]
	GMZB Bregs	CD19^+^GMZB^+^	GrzB	[133,134]
	CD9 Bregs	CD19^+^CD9^+^	IL-10	[135,136]
	Br1 Cells	CD19^+^CD25^+^CD71^+^CD73^lo^	IL-10 and Adenosine	[137,138]
	IgA^+^ Bregs	CD19^+^PD-L1^+^IgA^+^	IL-10 and PD-L1	[113]
	nBregs	CD19^+^CD5^+^CD23^-^	IL-10	[139,140]
Mouse	B10	CD19^+^ CD5^+^ CD1d^hi^	IL-10	[141]
	Marginal Zone B-cell	CD1d^hi^CD21^hi^CD23^−^IgM^hi^IgD^lo^	IL-10	[142]
	T2-MZP	B220^+^CD21^hi^CD23^+^IgM^hi^	IL-10	[143]
	TIM-1 Bregs	CD19^+^Tim-1^+^	IL-10 and IL-4	[128]
	Plasma	CD19^+^CD138^+^IgM^+^	IL-10 and IL-35	[144]
	Plasmablast	CD138^+^CD44^hi^	IL-10	[145]
	i35-Bregs	CD5^+^CD1d^hi^FcgIIb^hi^	IL-35	[146,147]
	Fas-L Bregs	CD19^+^CD5^+^FasL^+^	FasL and TGF-β	[148]
	PD-L1 Bregs	CD19^+^PD-L1^hi^	PD-L1	[149]
	GITRL Bregs	-	GITRL	[150]
	nBregs	CD19^+^CD5^+^CD23^-^	IL-10	[139,140]

## Data Availability

No new data were created or analyzed in this study.
